# Runx3, Brn3a and Isl1 interplay orchestrates the transcriptional program in the early stages of proprioceptive neuron development

**DOI:** 10.1371/journal.pgen.1011401

**Published:** 2024-12-23

**Authors:** Kira Orlovsky, Elena Appel, Shay Hantisteanu, Tsviya Olender, Joseph Lotem, Ditsa Levanon, Yoram Groner

**Affiliations:** Department of Molecular Genetics, Weizmann Institute of Science, Rehovot, Israel; University of California San Francisco, UNITED STATES OF AMERICA

## Abstract

**Background:**

The development and diversification of sensory proprioceptive neurons, which reside in the dorsal root ganglia (DRG) and express the tropomyosin receptor kinase C (TrkC), depend on the transcription factor (TF) Runx3. Runx3-deficient mice develop severe limb ataxia due to TrkC neuron cell death. Two additional TFs Pou4f1 (also called Brn3a) and Isl1 also play an important role in sensory neuron development. Thus, we aimed to unravel the chromatin state of early-developing TrkC neurons and decipher the Runx3 high-confidence target genes (HCT) and the possible cooperation between Runx3, Brn3a and Isl1 in the regulation of these genes.

**Methods:**

*Runx3* expression is driven by the gene proximal P2 promoter. Transcriptome analysis was conducted by RNA-seq on RNA isolated from heterozygous (P2+/-) vs. homozygous (P2-/-) TrkC neurons and differentially expressed genes (DEGs) were determined. Genome-wide occupancy of Runx3, Brn3a, Isl1 and histone H3 acetylated on lysine 27 (H3K27Ac) was determined using CUT&RUN. The landscape of Transposase-accessible chromatin was analyzed via ATAC-seq.

**Findings:**

The intersection of Runx3 genomic occupancy-associated genes and DEG data discovered 244 Runx3 HCT. Brn3a and Isl1 were found to bind to numerous genomic loci, some of which overlapped with Runx3. Most genomic regions bound by each of these three TFs or co-bound by them resided in distantly located enhancer regions rather than in gene promoters. In activated and suppressed neuronal Runx3 HCT, Runx3 cooperated mainly with Brn3a to regulate expression through distantly located enhancers. Interestingly, suppression of non-neuronal immune genes was mainly managed via Runx3 without Brn3a. The distribution of ATAC and H3K27Ac marked regions in Runx3 peaks containing at least one RUNX binding site (Runx3_RBS) revealed that while most promoter regions were marked by ATAC, a prominent fraction of intron/intergenic regions occupied by Runx3, Brn3a or Isl1 were unmarked by ATAC and/or H3K27Ac.

**Conclusions:**

These analyses shed new light on the interplay of Runx3, Brn3a, Isl1, and open chromatin regions in regulating the Runx3 HCT in the early developmental stages of TrkC neurons.

## Author summary

While the importance of Runx3, Brn3a and Isl1 TFs during early development and specification of DRG TrkC sensory neurons is well established, the genomic details of their molecular function in this process are not well understood, largely because of lacking details on their genomic occupancy. In the current study, we concentrated on characterizing the chromatin state of early-developing TrkC neurons and deciphering the genomic occupancy landscape of Runx3 and two other TFs, Brn3a and Isl1. These analyses and our RNA-seq data revealed 244 Runx3 HCT in the early developmental stages of TrkC neurons and the mode of the combined action of Runx3, Brn3a, and Isl1 in regulating these genes. We found that all three TFs bind to numerous genomic loci, some of which overlapped. Most genomic regions co-bound by these TFs resided in distantly located enhancer regions rather than in gene promoters. While most promoter regions containing Runx3_RBS peaks were marked by ATAC and H3K27Ac, a prominent fraction of Runx3, Brn3a, and Isl1 occupied regions in intron/intergenic regions were unmarked by ATAC and/or H3K27Ac. Our analysis revealed cooperation between Runx3 and mainly Brn3a in neuronal genes that are either positively or negatively regulated Runx3 HCT. Interestingly, suppression of non-neuronal immune genes is largely managed via Runx3 without Brn3a.

## Introduction

Proprioceptive neurons are essential for limb movement coordination, they reside in the dorsal root ganglia (DRG) and are marked by the tropomyosin receptor kinase C (TrkC) expression [1]. The TF Runx3 is crucial for the proper development of TrkC neurons [2]. In Runx3-deficient mice, TrkC neurons are generated but fail to extend central and peripheral afferents, leading to neuronal death and limb ataxia. However, the transcription program orchestrated by Runx3 at the early developmental stages of TrkC neurons is largely unknown. *Runx3* is expressed in TrkC neurons via the P2 promoter, and similarly to Runx3-deficient mice, P2-/- mice also develop severe limb ataxia [3]. Comparison between the E11.5 transcriptome of heterozygous (P2+/-) and homozygous (P2-/-) TrkC DRG neurons, revealed Runx3-responsive genes [3]. Still, it was unclear which of the differentially expressed genes were direct targets of Runx3. Thus, genomic landscape analysis of Runx3 binding in TrkC neurons in E11.5 DRG was also required to gain insight into the transcription program orchestrated by Runx3 at their early developmental stages. In the current study, we concentrated on characterizing the chromatin state of early-developing TrkC neurons and deciphering the genomic occupancy landscape of Runx3 and two other TFs, Brn3a and Isl1, known as important sensory neurogenesis regulators. These analyses and our RNA-seq data revealed 244 Runx3 HCT in the early developmental stages of TrkC neurons and the mode of the combined action of Runx3, Brn3a, and Isl1 in regulating these genes. We also determined the genome-wide distribution of ATAC and H3K27Ac marked genomic regions and their overlap with Runx3, Brn3a, and Isl1 bound regions containing their respective consensus TF binding sites (TF_TFBS).

## Results

### BAC-E-GFP recapitulates Runx3 expression in TrkC neurons from earliest developmental stages

**Fig 1:**
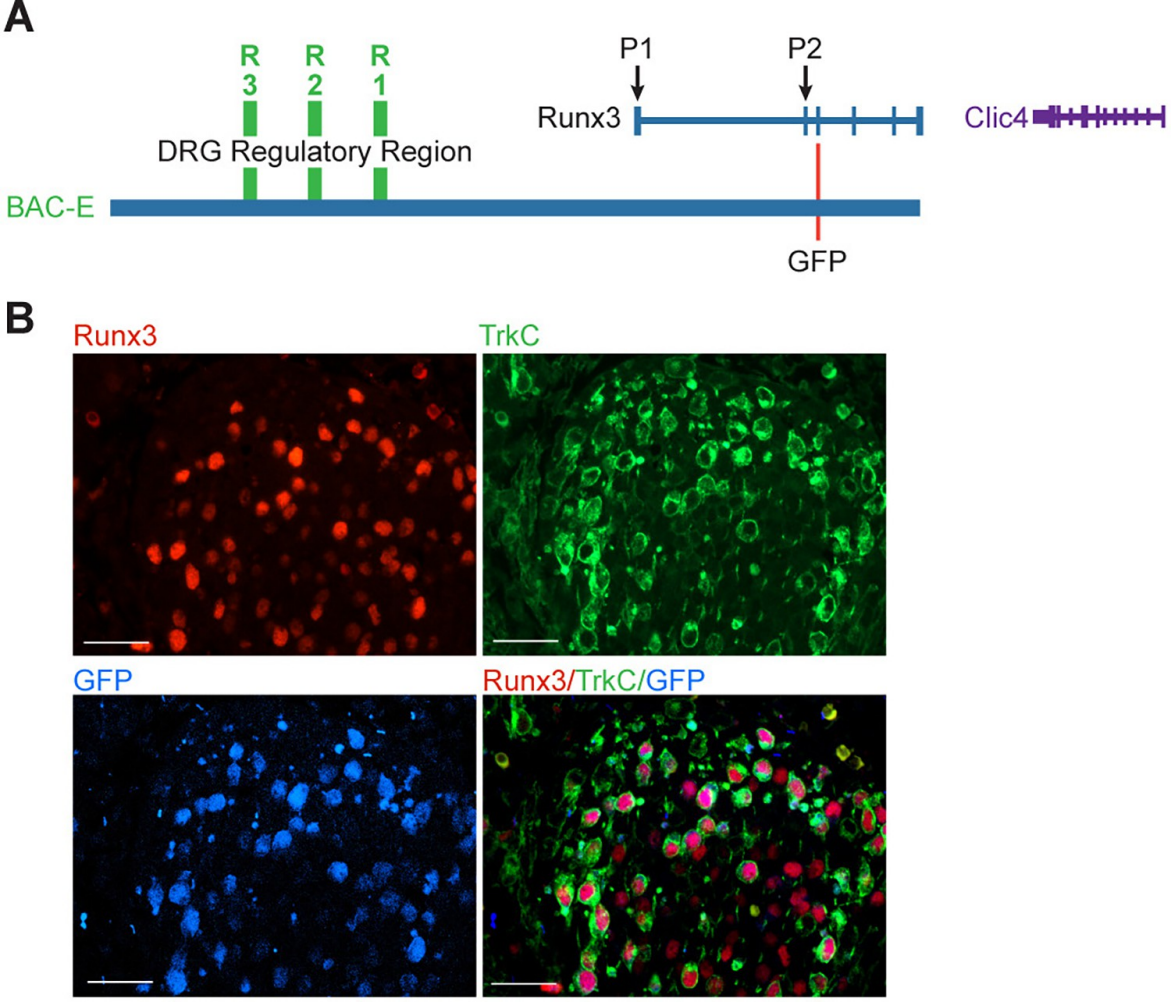
BAC-E-GFP recapitulates Runx3 expression in TrkC neurons from their early developmental stage. A, the *Runx3* locus including BAC-E that spans the regulatory region harboring the three *Runx3*-enhancer regions R1, R2, and R3. B, immunofluorescence of BAC-E-driven Runx3, TrkC, GFP, and merged in E11.5 DRG. Bar, 50μm.

Our previous study analyzed the pattern of reporter gene expression in the presence of reporter-expressing bacterial artificial chromosomes (BACs) that span the entire *Runx3* locus [3]. Among these, BAC-E (220 kb in length) that extends 5’ ~166 kb upstream of *Runx3* P1 promoter and 3’ reached the beginning of exon 6 (Fig 1A), conferred a high level of GFP expression in all Runx3-expressing tissues at E14.5, including DRG TrkC neurons [3]. BAC-E includes the three *Runx3* enhancers (R1, R2, and R3) shown to confer expression in TrkC neurons from the earliest neurogenic stages [3]. BAC-E-derived GFP is detected from early developmental stages of TrkC neurons at E11.5 (Fig 1B). Therefore, isolating Runx3 expressing TrkC neurons using transgenic mice expressing BAC-E-GFP is possible and informative.

### Accessible chromatin and H3K27Ac-marked regions in E11.5 Runx3-expressing DRG neurons

**Fig 2:**
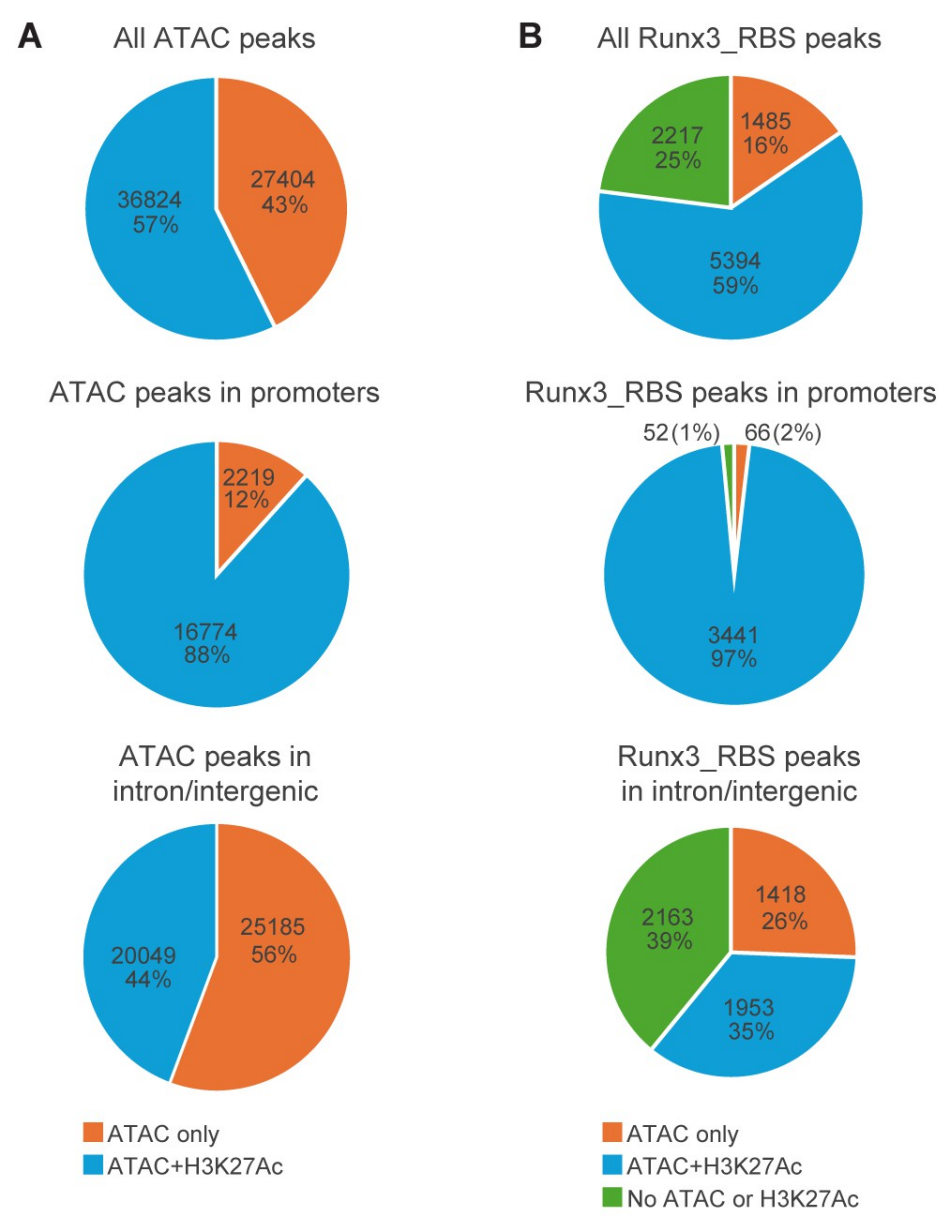
Distribution of ATAC and ATAC +H3K27Ac peaks in the whole genome and in Runx3_RBS peaks of E11.5 TrkC neurons. A, all ATAC peaks (top), ATAC peaks in promoters (middle) and ATAC peaks in -only intron/intergenic (bottom). B, all Runx3_RBS peaks (top), Runx3_RBS peaks in promoters (middle), and Runx3_RBS peaks in introns/intergenic regions (bottom).

We analyzed the chromatin state by ATAC-seq to get a first glance at accessibility in regulatory regions of TrkC neurons at this early (E11.5) differentiation stage. This analysis revealed ~64,000 regions of open chromatin, of which ~30% were in gene promoters [(up to ± 1 kb from the transcription start site (TSS)] and ~70% in intron/intergenic regions (Fig 2A). H3K27Ac marked genomic regions are well established as signatures of putative active transcriptional enhancers [4]; however, H3K27Ac also marks many promoter regions [5]. Using the CUT&RUN method on E11.5 TrkC DRG neurons, revealed 32,655 H3K27Ac-marked genomic regions, ~40% of which resided in promoter regions. Analyzing the extent of overlap between all ATAC and H3K27Ac-marked regions revealed that most (~88%) of ATAC peaks in promoters and ~44% of ATAC peaks in intron/intergenic regions were also marked with H3H27Ac (Fig 2A). This result could be explained, at least in part, by the temporal changes in chromatin accessibility that tend to precede acquisition of the H3K27Ac marker [6].

Genome-wide association of ATAC peaks with genes, using GREAT, revealed 4,557 genes with ATAC peaks only in their promoters (P-only), 5,431 genes with ATAC peaks only in their intron/intergenic regions (I-only), and 8,971 genes with ATAC peaks in both their promoters and intron/intergenic regions (P-I). We then determined whether these three gene groups differed in enriched GO terms. Interestingly, the P-I gene group was the only one prominently enriched for multiple neuronal terms under all GO term categories (S1 Table). The P-only genes showed minimal enrichment for some neuronal GO terms under “Cell component” (GO:CC) and KEGG. The I-only group of genes was mainly enriched for terms associated with immune/inflammatory processes, although a few neuronal terms were also enriched (S1 Table). Taken together, the identity of the E11.5 Runx3-expressing DRG neurons was most prominently observed in the P-I group of genes, which harbors ATAC peaks both in their promoters and intron/intergenic regions that contain genomic enhancers. Moreover, among the corresponding three H3K27Ac-marked gene groups, the P-I group and to a lesser extent the I-group revealed neuronal identity (S1 Table).

### Runx3-bound genomic regions in E11.5 TrkC neurons

The genome-wide landscape of Runx3 binding revealed 13,443 Runx3 peaks and 9,601 of them (71%) harbored at least one RBS (Runx3_RBS). The genomic distribution of Runx3_RBS peaks showed that ~38% resided in promoter and 62% in intronic/intergenic regions (Fig 2B). Association of these Runx3_RBS peaks with genes, using GREAT, revealed 2,776 genes with P-only peaks, 4,248 with I-only peaks and 1,356 genes with both (P-I). GO annotation enrichment analysis revealed prominent enrichment for neuronal terms in the P-I and I-only gene groups, whereas the P-only gene group showed minor enrichment for neuronal terms and prominent enrichment for general terms such as metabolic process and transcription (S1 Table). Notably, the proportion of I-only genes among all genes with Runx3_RBS-bound regions (51%) was much larger than that of I-only genes with ATAC or K3K27Ac peaks (29% and 18%, respectively). These results reveal that the Runx3_RBS peaks in intron/intergenic regions are mainly responsible for conferring the neuronal identity of the isolated cells. Of note, 2,638 out of the 4,248 genes in the I-only Runx3_RBS group did not overlap with those in the I-only ATAC or H3K27Ac groups. Moreover, these 2,638 I-only genes with Runx3_RBS peaks were enriched for neuronal terms, suggesting that they could represent early neuronal genes, which are not yet fully activated by the acquisition of open chromatin and the H3K27Ac mark in genomic regions harboring enhancers.

Next, to assess the chromatin accessibility and H3K27Ac marker in Runx3-bound regions, we determined the overlap of genomic Runx3_RBS-bound peaks with ATAC and H3K27Ac peaks. Almost all (98%) Runx3_RBS-bound promoter regions overlapped with ATAC, and H3K27Ac also marked 90% of them. Among Runx3_RBS peaks residing in intron/intergenic regions, only 26% overlapped with ATAC-only, and 35% were marked by ATAC and H3K27Ac (Fig 2B). These results indicated that the proportion of promoter regions marked by ATAC and H3K27Ac and overlap with Runx3_RBS (97%, Fig 2B) was larger than in all promoter regions (59%, Fig 2B). In contrast, the proportion of ATAC and H3K27Ac marked intron/intergenic regions overlapping Runx3_RBS was like that in all intron/intergenic regions. Of note, both the ATAC and H3K27Ac signals in Runx3-bound promoters and intron/intergenic regions were significantly stronger than in ATAC and H3K27Ac peaks in regions without Runx3 binding (p < 2.2e-16, Kolmogorov Smirnov test, determined on the non-logarithmic values; S1 Fig). These results suggest that Runx3 could increase the degree of chromatin “openness”, as known to be caused by pioneer TFs, reviewed in Zaret et al [7].

### Additional TFs collaborate with Runx3 in regulating gene expression

**Fig 3:**
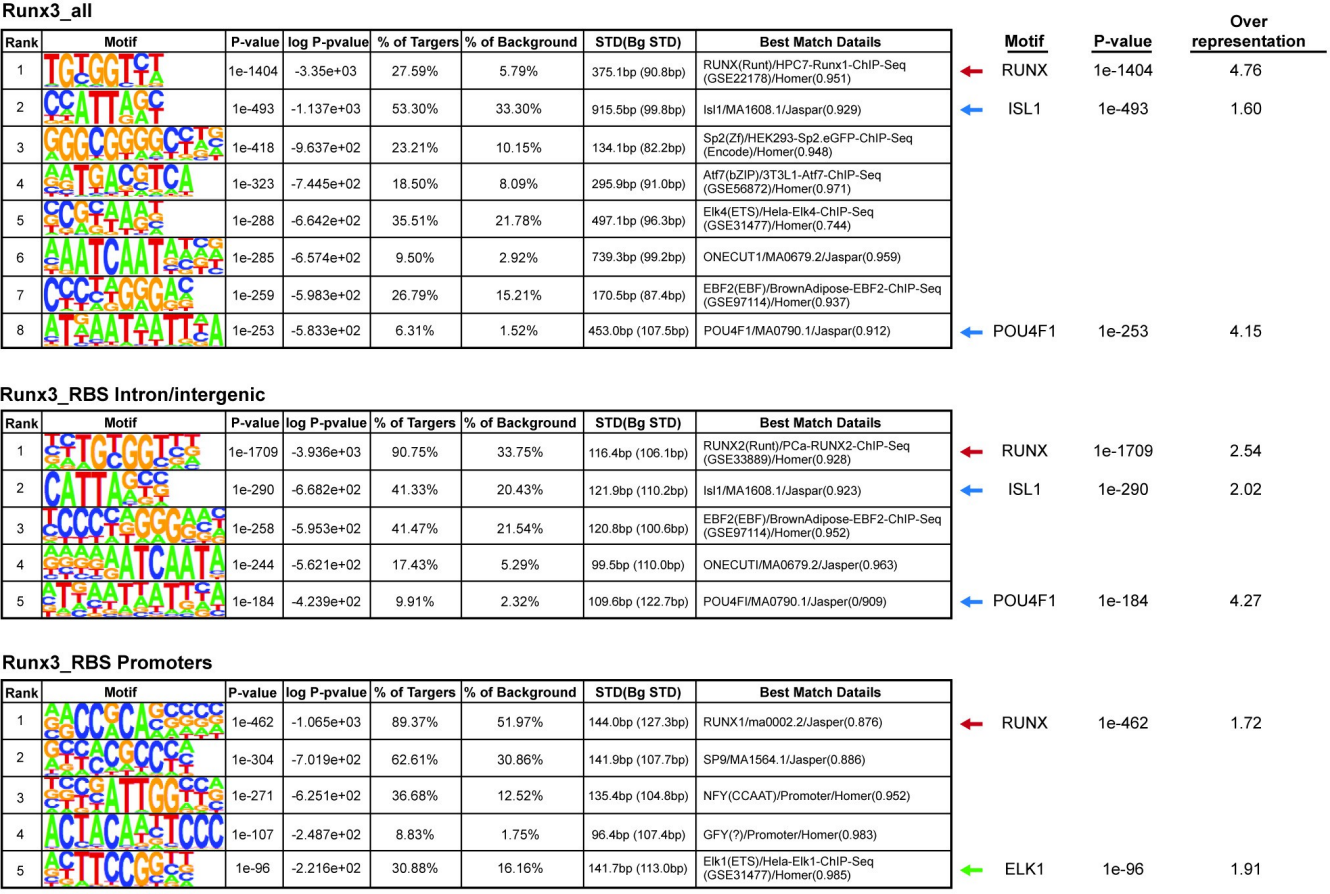
RUNX, ISL1 and POU4F1 are the most enriched TFBS motifs in Runx3 peaks residing in introns/intergenic regions but only RUNX is enriched in promoter regions. Homer de novo enriched motifs in all Runx3 and Runx3_RBS peaks.

Homer *de novo* TF motif enrichment analysis in Runx3 peaks revealed that RUNX was the most enriched motif. Moreover, the motifs for TFs essential for DRG neuron development, POU4F1/Brn3a and ISL1, were also enriched in all Runx3 peaks and Runx3_RBS peaks as well as in Runx3_RBS peaks residing in intron/intergenic regions (Fig 3). In contrast, promoter-bound Runx3_RBS peaks were enriched for RUNX and ELK1, a member of the ETS family, that is known to interact with RUNX family TFs [8], but were not enriched for POU4F1, ISL1, or other TFs that are known to be important for neuronal differentiation (Fig 3).

These results correspond with the finding that genes associated with Runx3 peaks in P-only regions were not enriched for neuronal GO terms, unlike those genes associated with Runx3-bound intron/intergenic regions. The results also suggested that Runx3 might cooperate with Brn3a and Isl1 in regulating gene expression in E11.5 TrkC neurons by binding to enhancer regions. The RNA-seq results supported this possibility, that showed high-level *Pou4f1/Brn3a* and *Isl1* expression in E11.5 TrkC neurons [3]. Furthermore, our and others’ studies have shown that Brn3a regulates the expression of *Runx3* and several other genes including *Cartpt*, *Dcc*, *Gal*, *Pirt*, *Piezo2* and *Scn7a* genes that are differentially expressed in Runx3-deficient neurons at early developmental stages of DRG neurons [3,9,10]. Similarly, Isl1 was shown to regulate the expression of *Cckar*, *Cbln2*, *Gal*, *Nova1 and Scn10a* in DRG neurons [11].

We conducted Brn3a and Isl1 CUT&RUN experiments and compared the genome-wide distribution of their bound regions to those of Runx3-bound regions to further explore the possibility that Brn3a and Isl1 cooperate with Runx3. The results revealed 32,823 Brn3a-bound and 16,036 Isl1-bound regions, of which 23,671 (72.1%) and 11,451 (71.4%) peaks, respectively, harbored their respective consensus motifs V$BRNF and V$LHXF (Genomatix sequence tools). However, while 38% of Runx3_RBS peaks resided in promoters, only 5% of Brn3a_BRNF and 4% of Isl1_LHXF peaks did so.

As expected, Homer *de novo* motif enrichment analysis (S2 Fig) confirmed that the most enriched motifs in Brn3a and Isl1-bound regions were POU4F1 and ISL1, respectively, corresponding to the Genomatix V$BRNF and V$LHXF TF family motifs, respectively. Of note, whereas the top 15 enriched motifs in Brn3a-bound regions included ISL1 and RUNX motifs, neither RUNX nor POU4F1 motifs were included in the top 15 enriched motifs in Isl1-bound regions (S2 Fig).

**Fig 4:**
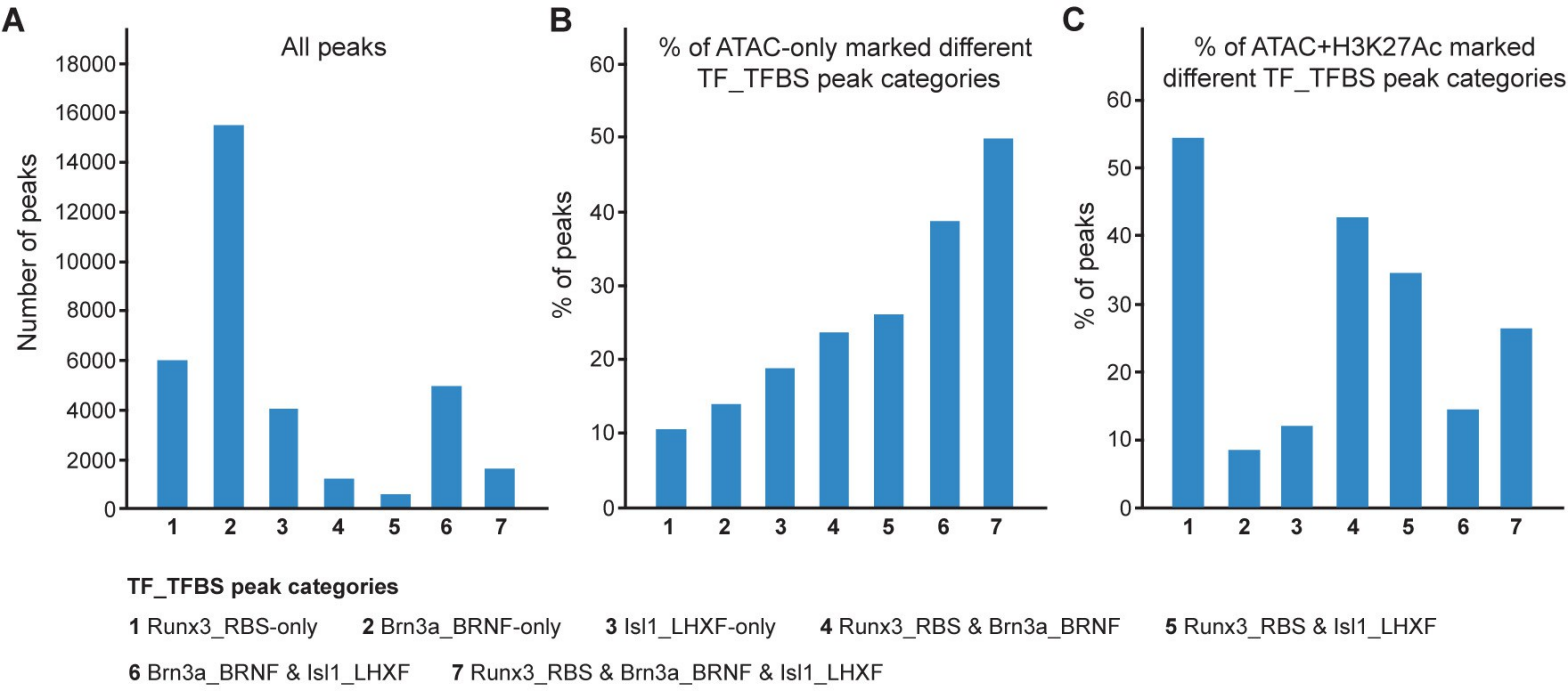
Whole genome content of the seven TF_TFBS peak categories and associated ATAC and H3K27Ac chromatin marks. TF_TFBS peaks were divided into 7 categories according to their overlaps with each other. Single peak categories of Runx3_RBS, Brn3a_BRNF or Isl1_LHXF peaks that do not overlap with each other (TF_TFBS-only) were marked as 1, 2, 3, respectively; Runx3_RBS peaks that overlap with Brn3a_BRNF or Isl1_LHXF were marked as 4 or 5; Brn3a_BRNF & Isl1 overlapping peaks without Runx3_RBS were marked as 6; and Runx3_RBS & Brn3a_BRNF & Isl1_LHXF overlapping peaks were marked as 7. A, number of all peaks in each TF_TFBS category. B and C, percent of peaks in each TF_TFBS category marked with ATAC-only or ATAC and H3K27Ac peaks, respectively.

Analysis of the peaks of all three TFs revealed that they can be divided into seven peak categories with various combinations of Runx3, Brn3a, and Isl1 TFs (Fig 4). Interestingly, overlap analysis between the three TF_TFBS peaks categories indicated that 63% of the Runx3_RBS-containg peaks and 66% of the Brn3a_BRNF-containing peaks did not overlap with the two other TF-bound peaks (Fig 4A, marked as “1 Runx3_RBS-only” and “2 Brn3a_BRNF-only”). Of all Isl1_LHXF-containing peaks, 4,080 peaks (36%), marked as “3 Isl1_LHXF-only”, did not overlap with the two other TF-bound peaks (Fig 4A). All three TF_TFBS categories overlapped in only 1,620 peaks, marked as “7 Runx3_RBS & Brn3a_BRNF & Isl1_LHXF” (Fig 4A). Furthermore, only 2,862 (30%) and 1,985 (21%) of the Runx3_RBS-containing peaks overlapped with Brn3a_BRNF and Isl1_LHXF-containing peaks, respectively (Fig 4A, marked as 4+7 and 5+7, respectively). Of the Brn3a_BRNF-containing peaks, 6,620 overlapped with Isl1_LHXF-containing peaks (marked as 6+7). The results indicated that while Brn3a and Isl1 peaks prominently overlapped, Runx3 overlapped with Brn3a more than with Isl1.

Overlap analysis of the three TF_TFBS peak types with ATAC marked chromatin regions revealed that 40–50% of regions with overlapping Brn3a_BRNF and Isl1_LHXF or Runx3_RBS and Brn3a_BRNF and Isl1_LHXF peaks (Fig 4B, marked as 6 or 7, respectively) were marked only with ATAC, while the rate was only 10–25% in other peak categories (Fig 4B). Interestingly, while ATAC and H3K27Ac marked 26–50% of Runx3_RBS-containing peaks (Fig 4C, marked as 1,4,5 and 7), they marked only 8–14% of Runx3-lacking peaks (Fig 4C, marked as 2,3 and 6). These results suggested that Brn3a and Isl1 might act as more efficient pioneer TFs than Runx3 by binding to inaccessible chromatin regions and initiating later chromatin opening.

**Fig 5:**
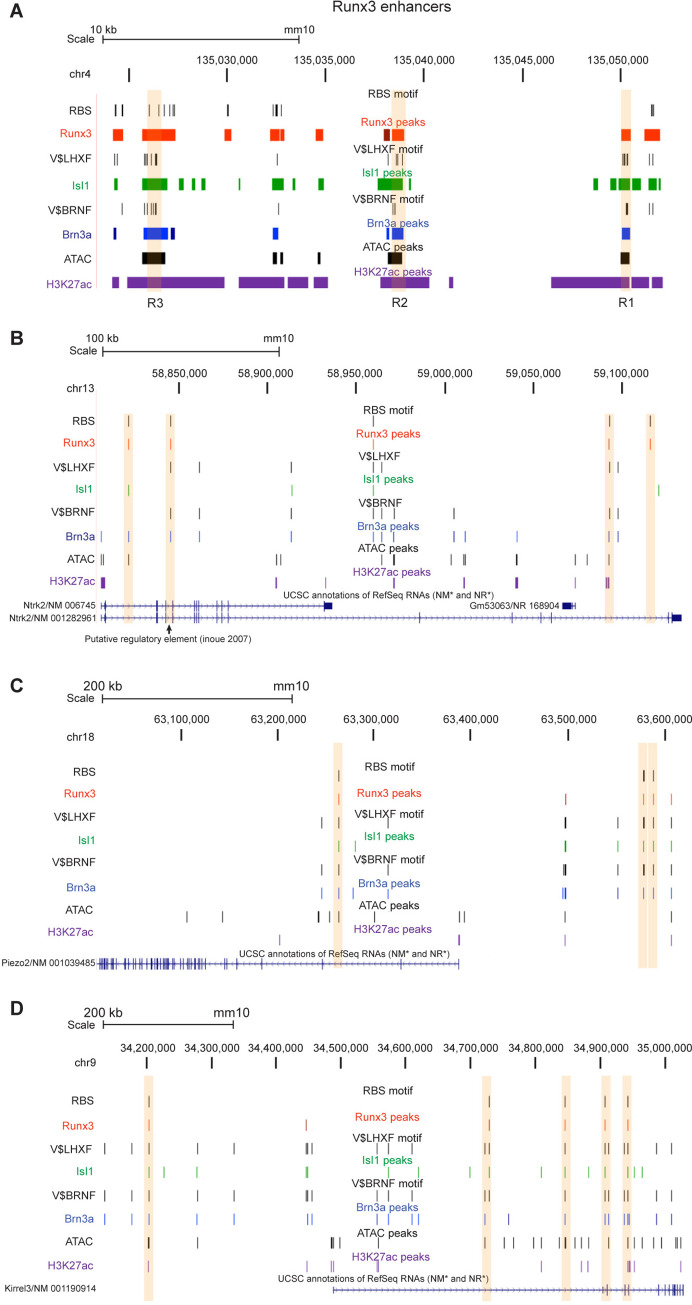
UCSC browser depicting the combined mode of binding of Runx3, Brn3a, and Isl1, and ATAC, and H3K27Ac marked regions in four of the Runx3 HCT. Runx3, Brn3a, and Isl1 peaks harboring their respective DNA binding sites, and ATAC and H3K27Ac are marked. Vertical pink bars mark the positions of Runx3_RBS peaks. A, Runx3 regulatory regions R1, R2 and R3. The *Runx3* P2 promoter is at chr4:135,151,494–135,152,493. B-D, *Ntrk2*, *Piezo2* and *Kirrel3*.

Brn3a and Isl1 TFs are expressed at the transition from neuronal precursors to post-mitotic TrkC neurons [12]. Brn3a suppresses the progenitor state and drives differentiation to the TrkC fate by activating genes required for TrkC neuronal functions and suppressing genes of other neuronal fates [10]. Based on the Brn3a-deficient phenotype, it was suggested that Brn3a precedes and is the principal regulator of Runx3 expression [10]. While Isl1 is also highly expressed in early developing Runx3-expressing neurons [3,11] and Isl1-deficient embryos show a delay in TrkC expression [11], it hardly affects *Runx3* expression and newborn mice do not display proprioceptive defects [11]. We have previously shown that *Runx3* expression is regulated by three highly conserved distant enhancers upstream of its P1 promoter (Fig 1). Two of these enhancers (R1 and R3) harbored Brn3a binding sites. Mutation in these sites markedly affected the activity of these enhancers [3]. These three enhancers offer good examples of Brn3a, Isl1, and Runx3 combined action. The genomic landscapes of Brn3a and Isl1 binding in TrkC neurons revealed that all three Runx3 enhancers (R1, R2 and R3) harbor overlapping Brn3a_BRNF and Isl1_LHXF peaks (Fig 5A), confirming their function as mediators of *Runx3* expression. Interestingly, the Brn3a_BRNF and Isl1_LHXF peaks in R3 overlapped with Runx3_RBS (Fig 5A). The R1 and R2 enhancers also bind Runx3. Still, they lacked RBS (Fig 5A). In addition, both Runx3_noRBS peaks in R1 and R2 enhancers are in chromatin-accessible “open” regions that are marked by ATAC and H3K27ac, which may enable binding of Runx3 to DNA in the absence of its canonical TFBS, possibly by physical interaction with Brn3a and isl1.

These results suggested a complex interaction and cooperation among the three TFs in regulating *Runx3* expression through these three enhancers. Furthermore, the genomic binding distribution of these three TFs suggested that Brn3a and Isl1 might also cooperate with Runx3 in regulating the expression of certain TrkC neuron genes by binding to the same or separate genomic region in these genes.

### Identification of high confidence Runx3 HCT

RNA-seq gene expression analysis was carried out on E11.5 isolated Runx3-expressing DRG neurons from heterozygous Runx3-P2^GFP/+^ and Runx3-deficient homozygous Runx3-P2^GFP/GFP^ mice. Using strict parameters (fold change ≥ 2, FDR <0.05), we identified 464 differentially expressed genes (DEGs) [3]. Under less stringent parameters for DEG identification (fold change ≥ 1.8, padj < 0.05), 625 DEGs were detected in the Runx3-deficient neurons, 150 DEGs were down-regulated and 475 were up-regulated.

We defined Runx3 HCT as DEGs that harbored at least one Runx3_RBS peak within their gene body (promoter, introns, 5’-UTR, or 3’-UTR) and/or in the upstream or downstream intergenic region that spans the distance between it and its neighboring RefSeq genes. We determined the genes corresponding to the 9601 Runx3_RBS peaks using Homer, which associates each peak with only one gene, and whose TSS is closest to the peak, and GREAT (default parameters). The latter assigns to each gene a regulatory domain consisting of a basal domain that extends 5 kb upstream and 1 kb downstream from its TSS and an extension up to the basal regulatory domain of the nearest upstream and downstream genes within 1 Mb. The Homer and GREAT analyses revealed 6,709 and 8,730 Runx3_RBS peak-associated genes, respectively. We then intersected these peak-associated genes with the 625 DEGs, which revealed 224 and 273 putative Runx3-regulated genes, respectively, but only 212 genes were common to both lists. Screening the genes not included among these 212 putative Runx3 targets indicated that many of their Runx3_RBS peaks resided within introns of neighboring non-DEGs. Therefore, we employed the UCSC genome browser, to which the Runx3_RBS peak coordinates were uploaded, to screen manually all 625 DEGs for the presence of Runx3_RBS peaks according to the parameters mentioned at the beginning of this section and identified the genes under direct Runx3 regulation. This screening revealed 244 Runx3 HCT, 86 down-regulated, and 158 up-regulated (S2 Table). Interestingly, the proportion of down-regulated Runx3 HCT among the down-regulated DEGs (86 out of 150 genes, 57.3%) was significantly higher than the proportion of the up-regulated targets among the up-regulated DEGs (158 out of 475 genes, 33.3%; Chi-squared statistic with Yate’s correction 22.0461; p < 0.00001, significant at p < 0.01; Fisher exact test statistic at p < 0.00001, significant at p < 0.01).

Recently, a single-cell RNA-seq study on E11.5 DRG identified several clusters of various cell types, two of which were identified as *Runx3*-expressing early and late proprioceptive neurons [13]. Therefore, it was interesting to determine which of our 86 down-regulated and 158 up-regulated Runx3 HCTs were in these proprioceptive neuron clusters. This assessment revealed that 76 out of the 86 down-regulated (88%) and just 24 of the 158 up-regulated (15%) Runx3 HCT were in the E11.5 proprioceptive neuron clusters. Most up-regulated Runx3 HCTs were not included in these proprioceptive or in the mechanoceptive neuron clusters (S3 Table). Interestingly, 64 out of the 158 up-regulated Runx3 HCT were expressed in clusters 5 and/or 6 at E10.5 prior to specification of proprioceptive or mechanoceptive neurons. These results confirmed that most detected down-regulated Runx3 HCT were indeed proprioceptive neuronal genes, whereas most of up-regulated Runx3 HCT were not expressed in either of these neuron subtypes, due to their suppression by Runx3. In line with these observations, the 86 down-regulated Runx3 HCT were enriched in GO annotation for neuronal terms. In contrast, the 158 up-regulated Runx3 HCT were enriched for immune system, cell death, and only marginally for neuronal-related terms (S3 Fig).

Next, we determined the presence of Runx3, Brn3a, or Isl1 peaks within promoters and intron/intergenic regions of the 244 Runx3 HCT and the distribution of ATAC and H3K27Ac peaks that overlap with these peaks. As described earlier for the genome-wide distribution of ATAC and H3K27Ac peaks, most Runx3 HCT promoter-residing peaks were marked by ATAC and/or H3K27Ac regardless of whether they harbored Runx3_RBS peaks. ATAC and/or H3K27Ac marked 60% of the Runx3-bound intron/intergenic peaks in Runx3 HCT. Brn3a-only peaks were the least marked by ATAC and /or H3K27Ac. The highest percentage of epigenetic marks in intron/intergenic peaks coincided with overlapping peaks containing Runx3, Brn3a, and Isl1 (S2 Table). These results suggested that Brn3a and Isl1 might collaborate with Runx3 in promoting chromatin accessibility in the Runx3 HCT, thereby regulating a significant proportion of them, either by shared binding to the same genomic region and/or by binding at separate genomic regions within these Runx3 HCT (S2 Table).

### Distinct properties of Runx3, Brn3a, and Isl1 peaks in down-regulated and up-regulated Runx3 HCT

**Fig 6:**
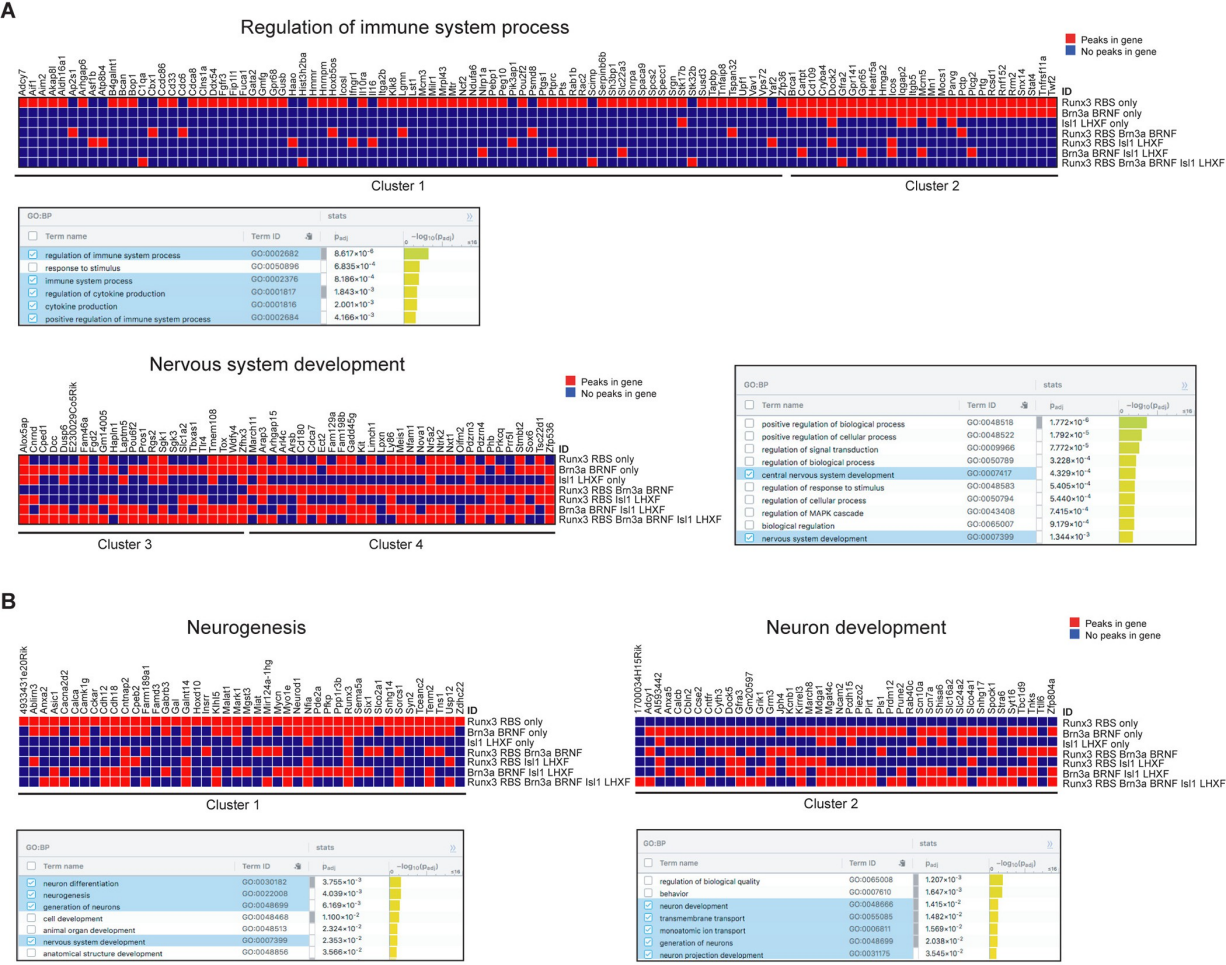
Up-regulated Runx3 target genes containing only Runx3_RBS peaks are enriched for immune functions while both up and down-regulated targets, including Runx3_RBS, Brn3a_BRNF, and Isl1_LHXF are enriched for neuronal functions. Clusters of Runx3 target genes according to the 7 categories of TF_TFBS peaks. A and B, up-regulated and down-regulated target genes, respectively, including the enriched GO annotation terms in each cluster. Red or blue boxes represent the presence or absence of each gene peak category.

To determine whether an association between TF peak categories in the Runx3 HCT and GO annotation exists, we performed K-means clustering (using R software) of the up-regulated and down-regulated Runx3 HCT according to their peak categories and subjected each cluster to GO annotation. Analysis of the up-regulated Runx3 HCT revealed that Cluster 1 consisted of 77 genes, including 55 harboring just Runx3-only peaks, and was enriched for immune system process terms. Cluster 2 included 27 genes that harbored mainly Runx3-only and Brn3a-only peaks, did not reveal any enriched GO terms, and contained similar numbers of genes with neuronal and immune system functions (Fig 6A). Clusters 3 and 4 comprised 54 genes, all containing Runx3, Brn3a, and Isl1 peaks and were enriched for neuronal terms (Fig 6A). The down-regulated Runx3 HCT formed two clusters enriched for Brn3a-only peaks. Cluster 1 also contained Runx3-only peaks, while Cluster 2 did not (Fig 6B). Genes in both clusters also contained Isl1 peaks and GO annotation analysis revealed enrichment for neuronal terms (Fig 6B). This clustering analysis revealed that the clusters in which most genes contained Runx3, Brn3a and isl1 peaks were enriched for neuronal terms. Taken together, these analyses indicated that Runx3 cooperates with Brn3a and Isl1 in activating or suppressing neuronal genes. In contrast, non-neuronal immune-related gene suppression was mainly done by Runx3, with minimal cooperation with Brn3a and Isl1.

### The role of Runx3 HCT in regulating proprioceptive neuron functions

**Fig 7:**
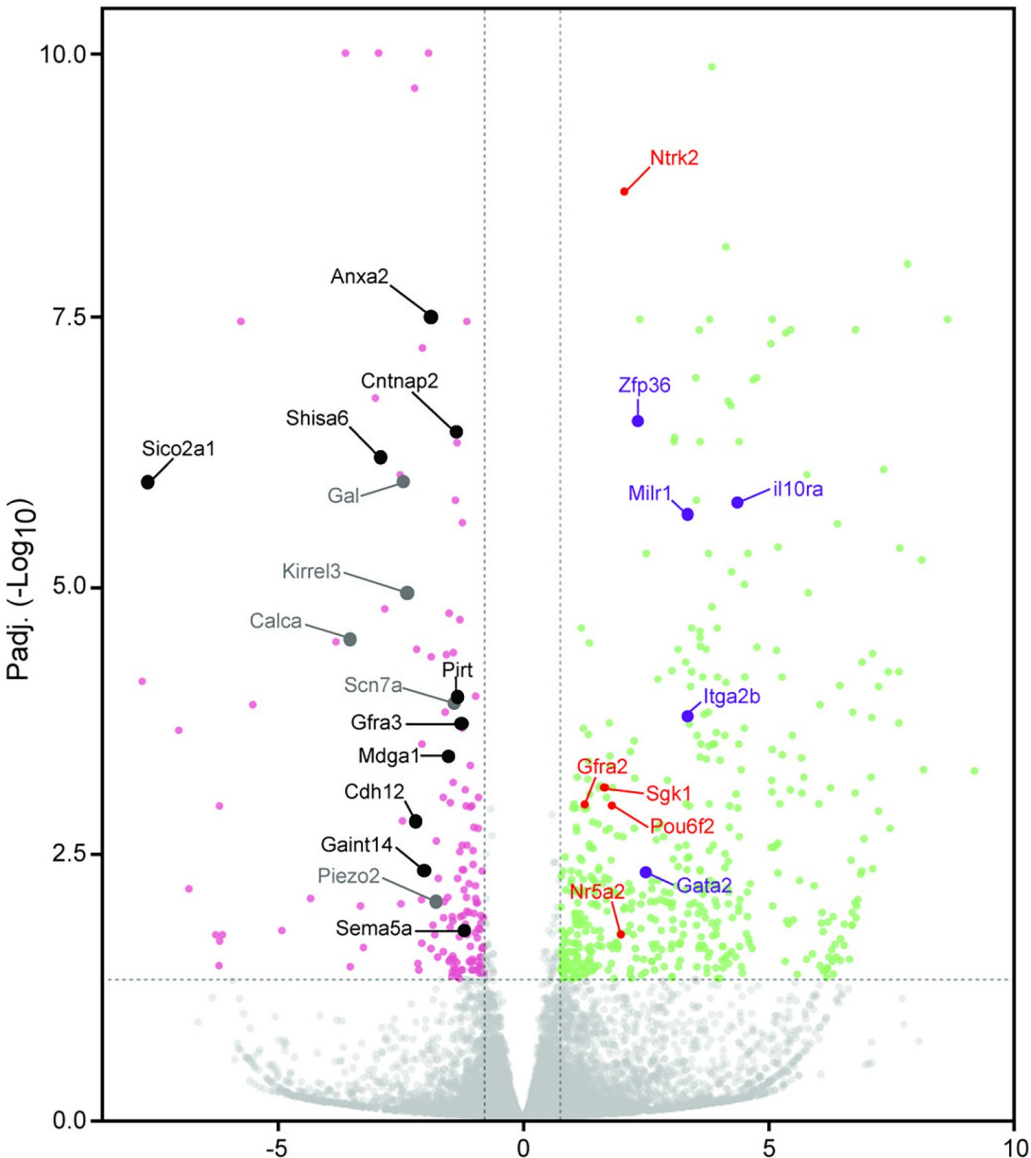
Runx3 dictates the TrkC neuron identity by negative regulation of TrkB mechanoceptive genes and immune genes and positive regulation of proprioceptive genes. Volcano plot of some down-regulated and up-regulated Runx3 target genes. Gene names outlined in black or gray, represent down-regulated neuronal genes expressed in proprioceptive or in both proprioceptive and mechanoceptive neurons, respectively. Gene names marked in red or purple, represent up-regulated neuronal genes expressed in mechanoceptive neurons or immune related genes, respectively.

Runx3 is known to act both as a transcriptional activator and as a repressor [14]. This dual mode of action fits very well Runx3 requirements during TrkC neuron diversification, namely, to allow the induction of gene-specific neuronal lineage and to shut off the expression of genes specific to previous stages and/or other cell types [12]. This phenomenon is illustrated in the Volcano plot (Fig 7), where it is clearly shown that immune genes and non-TrkC neuronal mechanoceptive genes are up-regulated in the absence of Runx3 whereas TrkC neuronal genes are down- regulated in the absence of Runx3.

One up-regulated target gene in Runx3-deficient cells was *Ntrk2*, which encodes for TrkB, and is normally expressed in TrkB but not in TrkC neurons. Runx3 is expressed in the pre-bifurcation proprioceptive/mechanoceptive neurons during early developmental stages. These neurons co-express the TrkB and TrkC surface markers of mechanoceptors and proprioceptors, respectively. As differentiation proceeds and these two lineages become distinct, Runx3 remains expressed only in TrkC proprioceptors [15].

In Runx3-deficient mice, the early hybrid TrkB/TrkC neurons fail to segregate into single TrkC neurons, indicating that Runx3 is crucial for repressing *Ntrk2* expression in cells destined to become TrkC neurons [16].

The resulting up-regulation of *Ntrk2* in Runx3-deficient cells reflects its function as a transcriptional repressor during TrkC neurons development. It was suggested that Runx3 binding to a putative regulatory element that contains a cluster of Runx-binding sites might be responsible for *Ntrk2* suppression in TrkC neurons [17]. We have confirmed the prediction that *Ntrk2* was a direct target of Runx3. Runx3-deficient E11.5 TrkC neurons express elevated levels of *Ntrk2* [3]. Furthermore, we found that Runx3 binds to five intronic regions in the *Ntrk2* gene (Fig 5B). One of these regions in intron 5, matches the putative regulatory element mentioned above. Moreover, *Ntrk2* expression induced by Brn3a [10] correlated with nine intronic Brn3a_BRNF peaks. Four of the Runx3_RBS peaks overlapped Brn3a peaks. It is of interest to determine whether these sites are involved in suppressing *Ntrk2* in TrkC neurons, while additional Brn3a-only peaks alone or in combination with Isl1 are responsible for *Ntrk2* activation. The most likely scenario is that during the early developmental stages of DRG TrkC neurons, Brn3a induces Runx3, and both TFs cooperate, possibly also with Isl1, to down-regulate *Ntrk2* expression. *Ntrk2* is not the only TrkB neuron-specific gene repressed by Runx3. Several other up-regulated Runx3 HCT, depicted in Fig 7 (*Gfra2*, *Nr5a2*, *Pou6f2*, and *Sgk1*), were also identified as mechanoceptive genes [13]. Therefore, suppression of the TrkB-specific genes mentioned above is part of the program by which Runx3 consolidates the TrkC neuronal lineage. As indicated above (S3 Fig), the up-regulated Runx3 HCT included genes with immune-related functions. Some of these genes (*Gata2*, *Il10ra*, *Milr1*, *Itga2b*, and *Zfp36*) are depicted in Fig 7. This finding indicated that suppressing these genes is an important Runx3 function, as it prevents inappropriate expression of immune-related genes during TrkC neuron development.

Runx3 is instrumental in inducing the expression of additional genes essential for the proper development and function of sensory neurons; some are selectively expressed in proprioceptive neurons, and some are not (Fig 7). One of the down-regulated genes in the absence of Runx3 was *Piezo2*, which encodes a mechanosensitive ion channel [18]. We have shown that *Piezo2* expression in Runx3-deficient E11.5 TrkC DRG neurons was down-regulated compared to Runx3-expressing neurons [3]. Moreover, studies in mice have shown that a mouse line that lacked *Piezo2* in its proprioceptive neurons (*Pvalb-Cre*:*Piezo2*^*f/f*^) had severely uncoordinated body movements and abnormal limb positions [19]. Deletion of *Runx3* in neurons (Wnt1-Cre/Runx3*f/f*; [20]) recapitulated the ataxia phenotype that occurred in Runx3-deficient mice [2], and was associated with severe skeletal scoliosis. Deletion of *Piezo2* in proprioceptive neurons (*Pvalb-Cre*:*Piezo2*^*f/f*^) but not in osteogenic (*Col1a-Cre*:*Piezo2*^*f/f*^) or chondrogenic (*Col2a-Cre*:*Piezo2*^*f/f*^) cells resulted in skeletal defects, including spine malalignment and hip dysplasia [21]. Furthermore, human PIEZO2 deficiency results in proprioception defects and hip dysplasia [22].

*Piezo*2 is also down-regulated in the DRG of Brn3a-deficient and Isl1-deficient embryos [10]. It was interesting to note in the present study that *Piezo2* was a Runx3 HCT with one Runx3_RBS peak in intron 2 that overlaps with Brn3a_BRNF and Isl1_LHXF peaks and resides in a chromatin-accessible region with ATAC and H3K27Ac markers (Fig 5C). Two other Runx3_RBS peaks, found in an intergenic region upstream of its TSS, overlapped with Brn3a_BRNF and Isl1_LHXF peaks, but not with ATAC or H3K27Ac (Fig 5C). *Piezo2* also harbored 3 additional Brn3a_BRNF and Isl1_LHXF overlapping peaks and three Brn3a_BRNF-only peaks, none overlapping with Runx3_RBS (Fig 5C). These findings can explain, at least in part, the ataxia and skeletal abnormalities seen in Runx3 and Piezo2-deficient mice.

*Kirrel3*, which encodes a synaptic adhesion molecule, was previously shown to be expressed as early as E11.5 in DRG TrkC neurons and was suggested to play a role in axonal pathfinding, cell recognition, and synapse formation of DRG neurons on appropriate target cells, including the targeting of proprioceptive neurons on muscle spindles through the interaction with Nephrin [23]. We have now found that *Kirrel3* harbored five Runx3_RBS peaks, four of which in introns and one in an intergenic region -280 kb from its TSS (Fig 5D). Four of the Runx3_RBS peaks in *Kirrel3* also overlapped with Brn3a_BRNF and Isl1_LHXF peaks. *Kirrel3* also harbored 13 separate Brn3a_BRNF and three Brn3a_BRNF_Isl1-LHXF overlapping peaks (Fig 5D).

These results suggested that Runx3 might cooperate with the highly expressed Brn3a and Isl1, in negatively regulating *Ntrk2* and positively regulating the expression of *Piezo2* and *Kirrel3* in DRG proprioceptive neurons already at the early stages of TrkC neuron development. However, while Kirrel3-deficient mice displayed certain sensory abnormalities such as autism-like and hyperactive behavior phenotypes, no ataxia was reported [24]. Therefore, although *Kirrel3* plays a role in proprioceptive neuron targeting on muscle spindles, even its complete loss is insufficient to induce the severe proprioceptive defects seen in Runx3-deficient mice.

### Additional Runx3 HCT harboring Runx3-bound regions lacking RBS

Our definition of Runx3 HCT considered only DEGs associated with Runx3-bound regions that harbored a consensus RBS. However, we realized that many of these Runx3 HCTs also harbored Runx3 peaks but no RBS (Runx3_noRBS). Moreover, whole genome analysis of Runx3 peaks revealed that 92% of the 3,842 Runx3_noRBS peaks reside in chromatin-accessible regions (ATAC-marked), and the genes associated with these Runx3_noRBS peaks were enriched for GO neuronal terms (S4 Table). Therefore, we searched for DEGs associated only with Runx3_noRBS peaks that could be regarded as Runx3 HCT in addition to the 244 Runx3 HCT mentioned in the previous subsection. To that end, we intercrossed the lists of genes with Runx3_noRBS peaks, the 244 Runx3 HCT and the 625 DEGs. This analysis revealed 37 such genes, 11 down-regulated and 26 up-regulated in Runx3-deficient neurons (S4 Table). Interestingly, of their 41 Runx3_noRBS peaks, 39 were marked by ATAC, and 25 overlapped with Brn3a and/or Isl1 peaks (S4 Table). All 11 down-regulated genes (*Adamts17*, *Ctdspl*, *Dio3*, *Faim2*, *Ndrg1*, *P4ha2*, *Pam*, *Pcbp3*, *Pcp4*, *Rgs4*, *and Slc6a15*) and five of the up-regulated genes (*Atxn10*, *Cst3*, *Mrpl34*, *Nwd2*, *and Rassf7*) were expressed in the proprioceptive DRG neuron clusters 7 and/or 9 and some also in the mechanoceptive clusters 10 and/or 11 [13].

*Ctss*, a Runx3_noRBS-containing up-regulated gene (S4 Fig), that was not expressed in the proprioceptive or mechanoceptive clusters, encodes cathepsin S, a papain-like cysteine protease known for its immune function in antigen presentation. It is now understood that Ctss, which is normally expressed in immune and microglia cells, has a role in activating itch and pain (nociception) in DRG neurons [25]. The nociceptive activity probably results from Ctss functioning externally as a signaling molecule that activates protease-activated receptors 2 and 4 members of the G-protein-coupled receptor family. *Ctss* is thus another example of a gene that is normally not expressed in DRG TrkC neurons due to repression by Runx3. The results also suggested that Runx3 might regulate these 37 genes by recruitment to the DNA through interaction with Brn3a and Isl1, bringing the total number of Runx3 HCT to 281.

## Discussion

Runx3, Brn3a, and Isl1 TFs play an important role in early developing DRG sensory neurons by maintaining their viability and promoting their differentiation. Brn3a and Isl1 expression precedes that of Runx3, and as differentiation advances, Brn3a induces Runx3 expression that continues to be expressed in the proprioceptive TrkC neurons. The interconnection of TrkC neurons with motoneurons forms the simplest and most ancient neuronal circuit known as the stretch reflex arc. Phylogenetically, the importance of these TFs in sensory neurons is reflected by the fact that their expression and function are also conserved through evolution. For example, the sea anemone *Nematostella vectensis Runx* homolog, predicted to act as Runx3 homolog, is expressed in the tentacles where the cnidocytes (mechano/chemoreceptor cells) reside [26]. Analysis of Brn3 homologs in the *Nematostella vectensis NvPOU4* revealed expression in cnidocytes and a subset of neural sensory ganglion cell types [27]. The Isl1 homolog in *Nematostella*, *Islet*, is expressed in a broad subset of neurons and analysis of the chromatin-accessible regions revealed enrichment for the Islet motif [28].

Deletion of either Runx3 or Brn3a in mice is associated with loss of TrkC neurons while deletion of Isl1 had a milder effect. Given the above-mentioned, it was interesting to determine the Runx3 interplay with Brn3a and Isl1 in the regulating Runx3 HCT.

**Fig 8:**
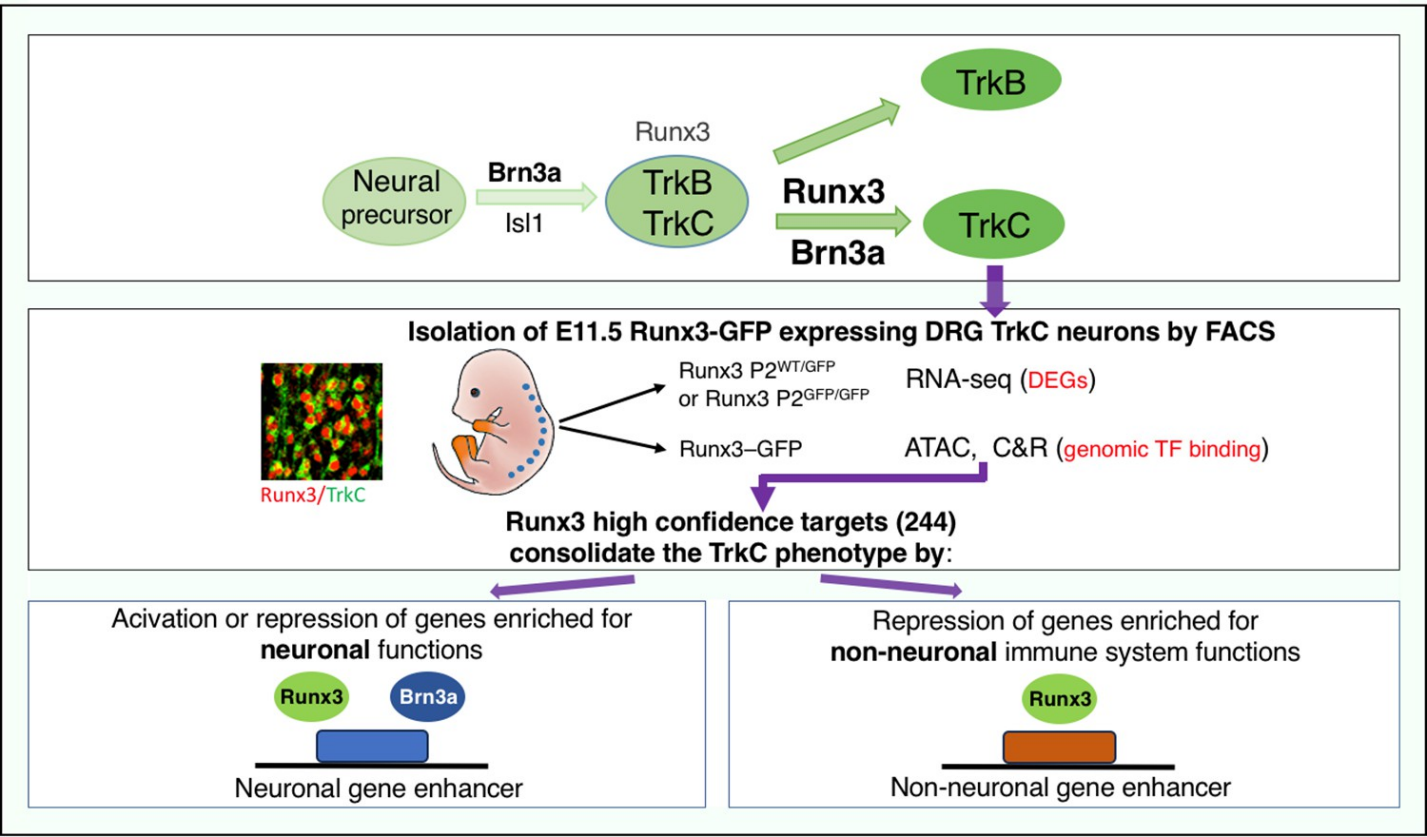
Summarizing scheme of the cooperation between Runx3, Brn3a and Isl1 in Runx3 HCT in early developing DRG TrkC neurons.

Our present experiments constitute the first description of the genomic binding landscape of Runx3, Brn3a, and Isl1 in early-developing DRG TrkC proprioceptive neurons. Compiled with RNA-seq data, we identified 244 Runx3 HCT, many of which were regulated by the combined activity of Runx3 and Brn3a and/or Isl1. Our analysis revealed cooperation between Runx3 and mainly Brn3a in neuronal genes that are either positively or negatively regulated Runx3 HCT. Interestingly, suppression of non-neuronal immune genes is largely managed via Runx3 without Brn3a (Fig 8).

The Runx3 HCT were DEGs that harbored Runx3_RBS peaks within their gene body and/or in the intergenic regions up to their nearest neighbors on both sides of the peak-bearing genes, acting as enhancers. However, it is known that enhancers of certain genes reside within the boundaries of their neighboring genes, such as the limb ZRS enhancer of *Shh* that resides within an intron of *Lmbr1* [29,30] or even when separated from the regulated gene by other genes [31–33]. In fact, GREAT associated Runx3_RBS peaks to genes identified ~30 DEGs in which the Runx3_RBS peaks resided within introns of their neighboring non-DEGs. Therefore, the identified 244 Runx3 HCT may be underestimated. Further studies, out of the scope of our present experiments, are needed to verify whether such Runx3_RBS peaks are indeed enhancers of genes whose expression was affected in Runx3-deficient TrkC neurons.

In addition to the 244 Runx3 HCT described above, we also detected 37 DEGs that harbored Runx3 peaks in genomic regions that lacked RBS (S4 Table). The presence of ATAC, H3K27Ac, Brn3a and/or Isl1 peaks in most of these 37 genes raises the possibility that Runx3 is recruited to these regions by Brn3a and/or Isl1 and might act with them to regulate the expression of these genes. To the best of our knowledge, no published biochemical evidence that Runx3 protein directly physically interacts with the Brn3a or Isl1 proteins exists. However, there is evidence in the literature that other POU, RUNX and ISL family members can physically interact (Oct1 interacts with Runx2 in mammary epithelial cells, [34] and the Brn3a ortholog UNC-86 interacts with the LIM-homeodomain TF MEC-3 in mechanosensory neurons in *C*. *elegans*, reviewed in [35]. Among the 244 Runx3 HCT, we identified up-regulation of *Ntrk2* and several other TrkB neurons-specific genes and down-regulation of many proprioceptive-specific genes in the absence of Runx3. Moreover, the Runx3 HCT up-regulated in Runx3-deficient TrkC neurons were enriched for immune system terms, suggesting that Runx3 suppresses the inappropriate expression of certain immune-associated genes in the developing TrkC neurons. Interestingly, a similar phenomenon of immune system genes up-regulation was described in neurons from the CK-p25 mouse model of neurodegeneration [36], in mice following brain hypoxic ischemia [37], in oligodendrocyte precursor cells of patients with multiple sclerosis [38] and in mouse hippocampal C1 neurons following contextual fear conditioning [39]. Of note, the 71 up-regulated genes in these hippocampal C1 neurons were also enriched for immune response terms [39], like the up-regulated Runx3 HCT. These findings indicated that Runx3 was important for maintaining the identity and fidelity of TrkC neurons by inhibiting the expression of certain genes that are normally expressed in other cell types, including other neuron subtypes and immune cells.

It is interesting to note that some Runx3 HCT encode for TFs that are either down-regulated (*Hoxd10*, *Mycn*, *Neurod1*, *Nfia*, *Prdm12* and *Zfp804a*) or up-regulated (*Gata2*, *Hoxb5os*, *Meis1*, *Nr5a2*, *Pou2f2*, *Sox6*, *Tox*, *Zfhx3* and *Zfp536*) in the absence of Runx3. It is possible that these TF Runx3 HCT may indirectly affect the expression of some of the DEGs, that did not qualify as direct Runx3 HCT due to a lack of Runx3_RBS peaks. It could also be suggested that some of these Runx3 target TFs could participate in regulating the expression of other Runx3 HCT.

The fraction of Runx3 peaks in promoters (42%) is much higher than that of Brn3a (11%) or Isl1 (5%) peaks. This trend was maintained in the peaks containing their respective consensus TFBS (38%, 5%, and 4% in Runx3_RBS, Brn3a_BRNF, and Isl1_LHXF, respectively). While 72% of Runx3_RBS peaks overlapped with ATAC peaks, only 35% and 49% of Brn3a_BRNF and Isl1_LHXF peaks, respectively, did so.

A prominent fraction of the intronic/intergenic Runx3, Brn3a, and Isl1 peaks lack ATAC marks, suggesting that at these sites they act as pioneer TFs. Isl1 and POU family TFs such as OCT4 were reported to have pioneering activity [40–42]. It was also shown that Pou4f3/Brn3c facilitates chromatin binding of Atoh1 TF and promotes mechanoceptor sensory neuron differentiation in inner ear hair cells and epidermal Merkel cells [43]. RUNX family members were suggested to be able to act as pioneer TFs that engage with condensed chromatin to facilitate its opening and promote the binding of other TFs [7]. Moreover, it was reported that Runx3 was required for chromatin accessibility in T cell receptor-activated CD8+ T cells [44]. Experiments that measured the ability of various TFs to bind to nucleosomes revealed that RUNX3 could bind to nucleosomes close to their ends, a different position than the binding of several POU domain TFs, including POU4F1 (BRN3A) and ISL1 [45] a property ascribed to so-called “pioneer TFs”. RUNX1 and RUNX2, the other RUNX TF family members, also bind to nucleosomes [45] and Runx2 was reported to regulate chromatin accessibility in neonatal osteoblasts [46]. In addition, we found that Runx3-bound genomic regions had a stronger ATAC signal than regions lacking Runx3 binding and that the fraction of ATAC/H3K27Ac-marked Runx3 HCT among Runx3, Brn3a and Isl1 overlapping peaks was larger than in regions bound by each of these TFs alone. While these results might imply that these three TFs cooperate to increase chromatin accessibility at their bound regions in TrkC neurons, we cannot dismiss the possibility that their joint binding prefers already open genomic regions.

It is currently unknown which of the intron/intergenic regions that bind Runx3, Brn3a, or Isl1 in the Runx3 HCT likely acting as enhancer regions, confer expression specificity to DRG TrkC neurons. It will be interesting to use transgenic mouse experiments to determine whether such putative enhancer elements, especially those that bind all three TFs, confer specific LacZ/GFP reporter expression in DRG TrkC neurons. It will also be interesting to determine whether the presence of H3K27Ac marks in such combined TF-bound enhancer elements is required for this DRG expression.

## Methods and materials

### Ethics statement

The experiments were strictly following the recommendations of the US National Institutes of Health Guide for the Care and Use of Laboratory Animals. The Weizmann Institute of Science Committee approved the Ethics of Animal Experiments protocols.

#### Mouse strains

Using recombineering, BAC-E (CHORI RP23-307D6) was modified to express GFP, and transgenic mice expressing the modified BAC (BAC-E-GFP) were generated [3]. These mice were with a Cb6F1 background. EGFP was fused into *Runx3*’s exon3, which appears in all functional products of Runx3.

#### Immunofluorescence

Runx3, TrkC, and GFP in DRGs of E11.5 BAC-E/GFP transgenic mice were detected as previously described [3].

#### DRG preparation

DRGs from 10–20 E11.5 embryos (mainly cervical) bearing BAC-E-GFP were dissected into cold Neurobasal A medium (Gibco 10888–022) containing 10% FCS and pooled together for each experimental replicate sample. For tissue dissociation, DRG were incubated in Eppendorf tubes for 13 min at 37°C and shaking at 700 rpm in 1 ml containing 2.5mg Trypsin B (Biological Industries, 03-046-1B) and 0.1% collagenase H (Roche Diagnostics, 11074032001) per embryo. The digested tissue was then centrifuged at 1300 rpm (200xg) for 7 min at 4°C, washed in 1ml Neurobasal A/10% Fetal Calf Serum (FCS) and resuspended in 300 μl ice cold Neurobasal-A medium, followed by passage through a 70 μm filter until complete tissue dissociation.

#### Isolation of GFP^+^TrkC^+^ neurons

The GFP^+^ TrkC^+^ neurons were sorted into DNase-free tubes containing 300 μl of FCS using FACS Aria SORP, with a 130 μm nozzle. Sorted cells were spun at 1300 rpm for 15 min, and supernatant was removed, leaving 100–200 μl. The resuspended pellet was transferred to 0.2 ml tubes (not low binding tubes), the cells were spun again as described above, and the supernatant was removed, leaving 10–20 μl to cover the pellet. The average number of isolated E11.5 embryo TrkC neurons per embryo was in the range of 20,000–30,000. In two experiments, DRG TrkC^+^ neurons from GFP^+^ or WT mice were isolated by magnetic-activated cell separation (MACS). DRG cells were incubated on ice for 60 min with biotin-conjugated rabbit anti-TrkC antibody (R&D systems BAF1404, 1:50), washed with Neurobasal-A medium and then incubated with streptavidin-conjugated magnetic nanoparticles (BD Bioscience, BDB557812) for 30 min at 8°C. The mixture of cells and nanoparticles was then placed on a magnet for 10 min, the supernatant containing unbound cells was carefully removed, and nanoparticle-bound cells were recovered after the removal of tubes from the magnet and resuspension in Neurobasal-A medium.

#### Assay for transposase-accessible chromatin (ATAC-seq)

The ATAC-seq protocol was modified from Buenrostro et al [47]. GFP^+^ cells (50,000) were sorted into an Eppendorf tube (not a low binding one) containing 100 μl of MACS buffer [1xPBS, 2mMEDTA, 1% BSA and protease inhibitors (Pi) mix (cOmplete Mini, Roche, 1:7)]. After removing the buffer by spinning at 1300 rpm for 10 min, the pellet was resuspended in 25 μl cold Lysis buffer (10mM Tris-HCl pH7.4, 10mM NaCl, 3 mM MgCl2, 0.1% IGEPAL CA-630) by pipetting 5 times, followed by spin at 500g for 20 min at 4°C. The pellet was kept on ice and resuspended in 12.5 μl Nextera 2xTD buffer, 2 μl Nextera Tn5 enzyme (Illumina Cat #FC-121-1030) and 10.5 μl H_2_O and incubated at 37°C for 1h with shaking (500 rpm). Cleaning the reaction was performed by adding 5 μl clean-up buffer (900mM NaCl, 30mM EDTA), 2 μl 5% SDS and 2 μl Proteinase K (20mg/ml) followed by incubation for 30 min at 40°C while shaking at 500 rpm. DNA fragments obtained from the ATAC reaction were purified using 2.2xAMPure beads (Beckman Coulter A63881), and then a barcoded library was prepared of using a modified protocol of Lara-Astasio et al. [48]. Nine PCR enrichment cycles were performed for ATAC-seq. One μl of library preparation was used for DNA quantification on Qubit, and 1 μl was analyzed by TapeStation. A pool of 4 nM DNA samples was prepared and 1.8 pM was loaded for sequencing using NextSeq 550 sequencing system with NextSeq 500/550 mid-output kit of Illumina (20024904). Three experimental replicates were carried out.

#### CUT&RUN

CUT&RUN protocol for neuronal cells was adopted from Skene PJ et al [49]. Twelve μl Concanavalin A magnetic beads (Bangs Laboratories, BP531) were washed twice with binding buffer (20mM HEPES-KOH, pH7.9; 10mM KCl, 1mM CaCl_2_, 1mM MnCl_2_), resuspended in 12 μl of the same buffer, added to a sample with 100,000–150,000 sorted cells in 500 μl cold Wash buffer (20mM HEPES, pH7.5; 150mM NaCl, 0.5mM Spermidine, 2mM EDTA, 0.1% BSA, 0.05% digitonin, Pi) and rotated for 6 min at RT. Beads were spun down and resuspended in 100 μl Ab buffer (10 μl 0.1M EDTA, 5 μl 10%BSA, 5 μl 5% Digitonin, 480 μl Wash Buffer). Rabbit anti-Runx3 (in- house produced 1:200) or anti-Brn3a (1:200, generously provided by Dr. E. Turner, eric.turner@seattle chilrens.org, 1:200) or anti-Isl1 (Abcam ab20670, 1:50) or anti- H3K27Ac (Abcam ab4729, 1:50) or non-immune rabbit serum used as control were added to the cells (final volume 500 μl) and tubes were rotated overnight at 4°C. The Tubes were placed on a magnet (for 2 min) and supernatant was replaced by 1 ml Wash buffer (20mM HEPES, pH7.5; 150mM NaCl, 0.5mM Spermidine, 0.05% digitonin, Pi). Cells attached to beads were washed twice, the tubes were removed from the magnet and cells were incubated with 150 pg Protein A conjugated Micrococcal Nuclease (pA-MNase) followed by rotation for 1 hr at 4°C. The samples were washed twice with the same wash buffer and incubated for 5 min on wet ice. 2 mM CaCl_2_ was added, and the samples were incubated for 30 min on ice. Reaction was stopped by adding 100 μl 2xSTOP buffer (0.65M NaCl, 20mM EDTA, 4mM EGTA, 0.05% digitonin, 10 μg/ml RNase A, 50 μg/ml glycogen, 0.004 pg/ml heterologous spike-in DNA) and the tubes were incubated for 10 min at 37°C. Tubes were placed again on the magnet, the supernatant was collected, and the DNA was extracted by phenol/chloroform. DNA fragments obtained from the CUT&RUN reaction were purified using 1.7xAMPure beads (Beckman Coulter A63881), followed by the preparation of a barcoded library using a library prep kit (NEBNext Ultra II DNA E7645S). PCR enrichment was performed for 12–13 cycles. Library preparation analysis and sequencing (1 μl for each sample) were conducted as above. The number of CUT&RUN experimental replicates were 8 of Runx3, 2 of Brn3a, 3 of Isl1 and 5 of H3K27Ac.

### Bioinformatics

#### ATACseq data

Reads were trimmed of their adapter using Cutadapt and aligned to mm10 genome (GRCm38.p5) with Bowtie2 (version 2.3.4.1) [50]. PCR duplicates were then removed using Picard (v 1.119). Non-uniquely mapped reads were filtered out with Samtools using the flags ‘-F 4 -f 0x2’. Nucleosome-free read pairs with inner distance of up to 120 bp were then selected from the alignment for downstream analyses. MACS2 (version 2.1.1.20160309) [51] was applied for peak calling with the setting: -f BAMPE—SPMR -B–Nomodel. The strength of the ATAC and H3K27Ac signal was calculated in RPKM using Deeptools, and statistical significance between different sets of peaks was calculated by the Kolmogorov Smirnov test. The custom code used for analysis of ATAC-seq data is deposited in GitHub and is accessible at https://github.com/TsviyaOlender/atac-seq-pipeline.

#### CUT&RUN data

Reads were trimmed from their adapter using Cutadapt following an alignment to the mm10 genome using ‘—no-mixed—no-discordant’. After alignment, fragments of ≤ 90 bp were selected for peak calling. Peak calling was performed with MACS2 against the control samples using ‘—keep-dup all’. The custom code used for analysis of CUT&RUN data is deposited in GitHub and is accessible at https://github.com/TsviyaOlender/cut_run

### Generating consensus and annotation of peaks from replicate experiments

We generated a consensus set of peaks from the biological replicates for every TF, ATAC, and H3K27Ac. This was done by selecting peaks that are shared by at least 50% of the experiment replicates (with ‘Bedtools intersect–F 0.5’). The consensus peaks were scored as those that appeared in 4/8 replicates for Runx3; 2/3 for ATAC; 3/5 for H3K27Ac; 2/3 for Isl1; and 2/2 for Brn3a. Because some CUT&RUN experiments showed contamination by sequences of undetermined origin such as (TA)n [52], peaks overlapping by >60% with repeat element regions (file RepeatMasker_rmsk_mm10.bed of UCSC) were removed. Blacklist peaks of Ensembl were removed from all datasets.

*De novo* enrichment analysis of DNA-binding motifs in TF-bound peaks was performed with Homer and identification of Runx3 binding sites (RBS) in consensus Runx3-bound peaks was done with a custom script designed to identify each all eight alternatives of the consensus RUNX motif RCCRCA (R = A/G), hereafter referred to as Runx3_RBS. We also used Genomatix to annotate Brn3a and Isl1 and their respective DNA-binding motifs (V$BRNF and V$LHXF).

Peak association to genes and annotation were performed with Homer (version 4.11) [53] and with GREAT version 4.04 analysis under default parameters [54]. Gene ontology (GO) annotation enrichment analysis was determined by gProfiler [55].

### Determination of overlap between different TF-bound (TF_TFBS) peak categories with or without ATAC and H3K27Ac chromatin marks

The consensus peaks of Runx3_RBS, Brn3a_BRNF, Isl1-LHXF and H3K27Ac were extended to 300 bp, ATAC peaks were extended to 500 bp and the overlap was determined using ‘Bedtools intersect’.

### K-means clustering of different peak categories in Runx3 target genes

The up-regulated and down-regulated Runx3 HCT were clustered based on the seven different TF peak categories using the pheatmap package in R with kmeans = 4 for the up-regulated target genes and kmeans = 2 for the down-regulated ones. All other parameters used the default values. The Morpheus tool (https://software.broadinstitute.org/morpheus) was applied to generate heatmaps based on the R clusters output genes.

## Supporting information

S1 Fig ATAC or H3K27ac signal is stronger in Runx3-bound regions compared to Runx3-unbound regions. Representative signal intensity of ATAC and H3K27ac peaks in Runx3-bound and unbound promoter and intron/intergenic genomic regions. a and b, boxplot (top) and schematic representation (bottom) of signal intensity distribution at -2 to +2 kb from the peak summit (bottom).
(TIF)

S2 Fig Top 15 Homer *denovo* enriched motifs in Brn3a and Isl1 bound genomic regions. (TIF)

S3 Fig Enriched GO annotation terms in down-regulated and up-regulated Runx3 HCT. (TIF)

S4 Fig UCSC browser derived genomic positions of Runx3, Brn3a and Isl1 peaks and ATAC and H3K27ac marked regions in *Ctss*. The pink vertical rectangle represents the position of a Runx3-bound region that lacks the RBS motif.
(TIF)

S1 Table GO terms enrichment in genomic regions of genes with ATAC, H3K27Ac and Runx3 peaks. Promoters-only, intron/intergenic regions-only or in promoters and intron/intergenic regions.
(XLSX)

S2 Table List of Runx3 HCT containing separate and overlapping Runx3, Brn3a and Isl1 peaks. (XLSX)

S3 Table Runx3 HCT expressed in clusters of DRG proprioceptive, mechanoceptive or other neuron subtypes in early embryonic development. (DOCX)

S4 Table Runx3_noRBS peaks with ATAC mark, GO terms enrichment of Runx3_noRBS-assocaiated genes, and 37 corresponding Runx3_noRBS HCT. (XLSX)
