# Supplementary figures and images for: Runx3, Brn3a and Isl1 interplay orchestrates the transcriptional program in the early stages of proprioceptive neuron development
Source: PLoS Genet. 2024 Dec 23;20(12):e1011401. doi: 10.1371/journal.pgen.1011401 (PMC11729954; doi:10.1371/journal.pgen.1011401)

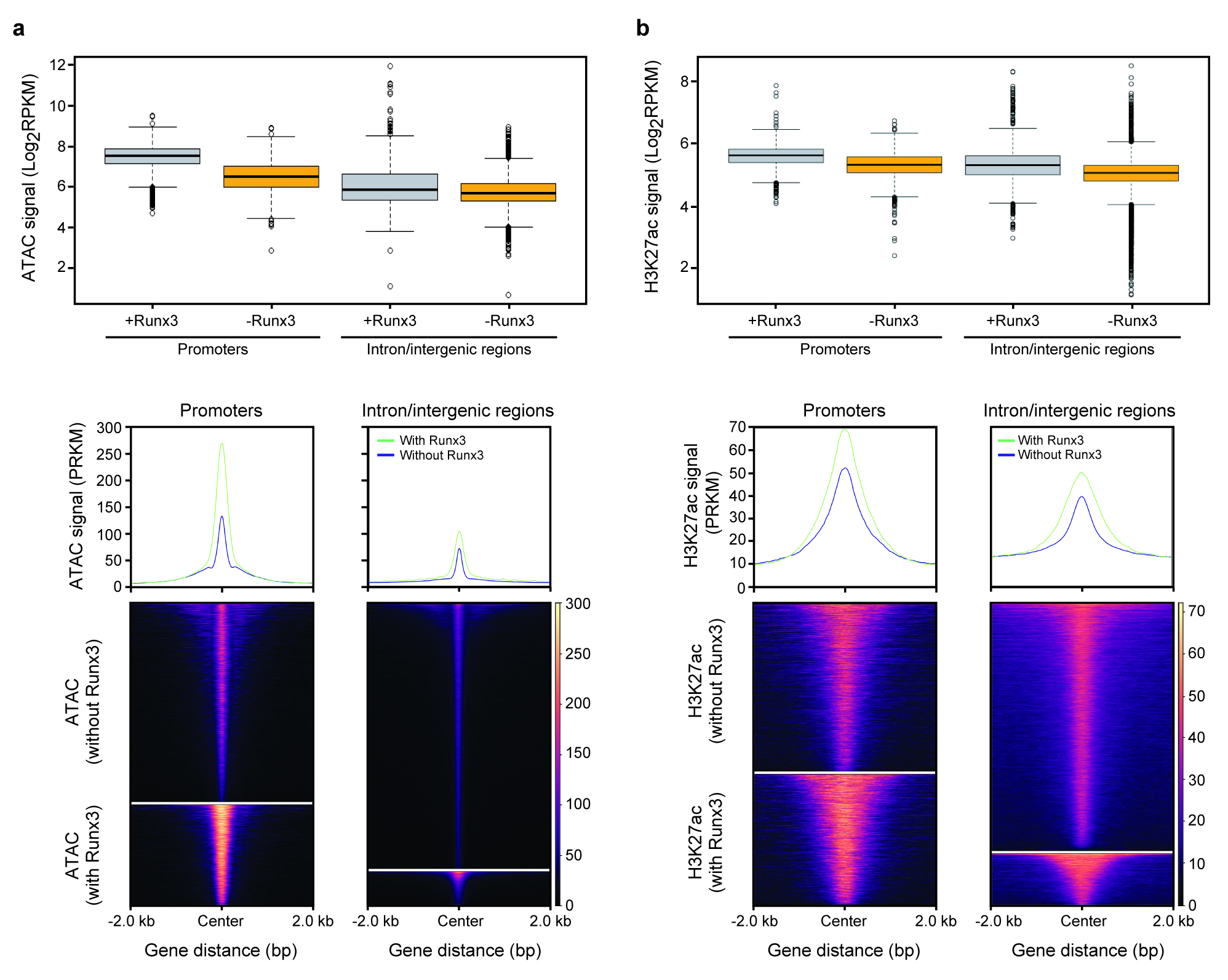

Supplement: S1 Fig — Representative signal intensity of ATAC and H3K27ac peaks in Runx3-bound and unbound promoter and intron/intergenic genomic regions. a and b, boxplot (top) and schematic representation (bottom) of signal intensity distribution at -2 to +2 kb from the peak summit (bottom). (TIF) [file pgen.1011401.s001.tif]

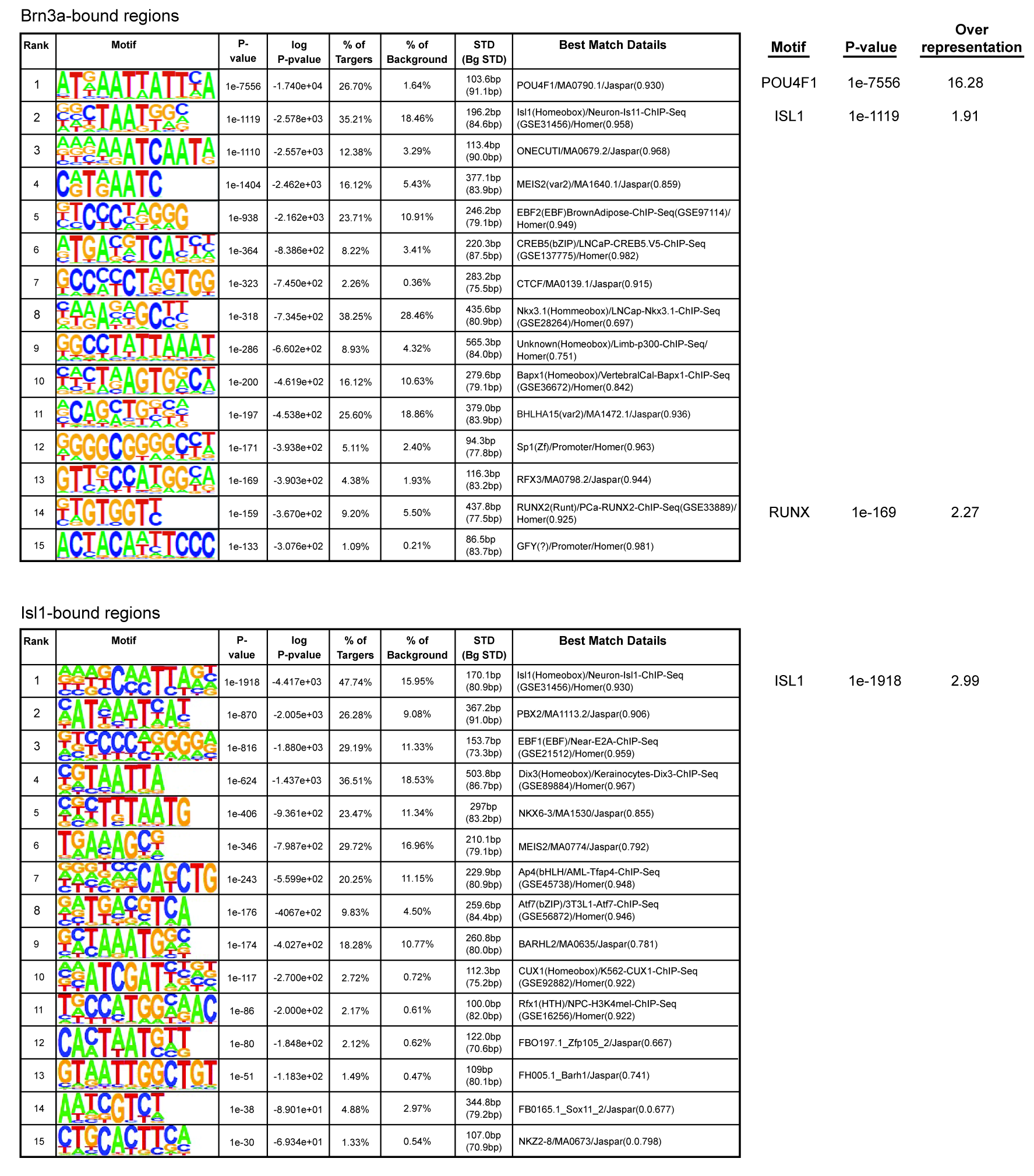

Supplement: S2 Fig — (TIF) [file pgen.1011401.s002.tif]

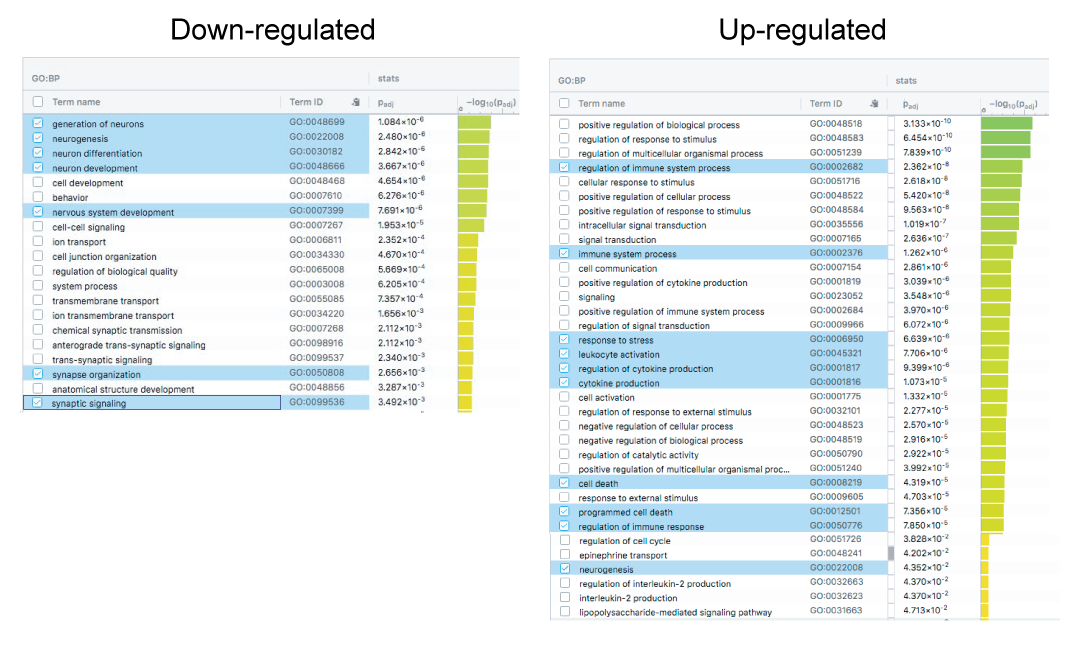

Supplement: S3 Fig — (TIF) [file pgen.1011401.s003.tif]

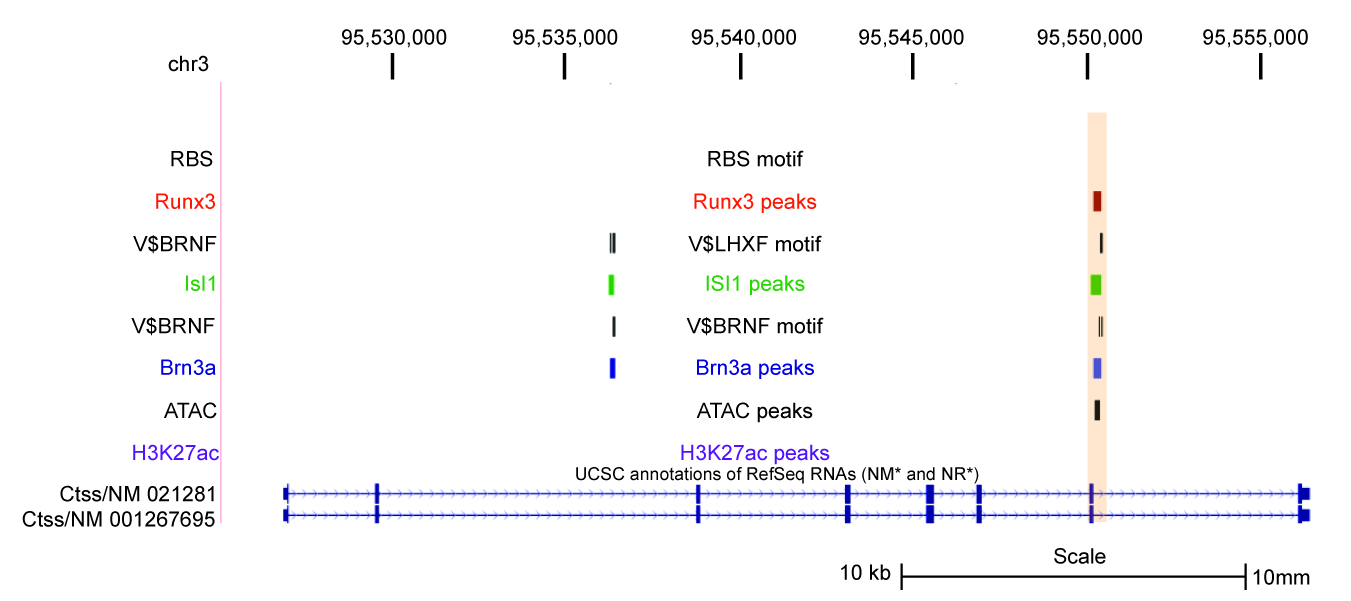

Supplement: S4 Fig — The pink vertical rectangle represents the position of a Runx3-bound region that lacks the RBS motif. (TIF) [file pgen.1011401.s004.tif]
