# Supplementary material for: Runx3, Brn3a and Isl1 interplay orchestrates the transcriptional program in the early stages of proprioceptive neuron development
Source: PLoS Genet. 2024 Dec 23;20(12):e1011401. doi: 10.1371/journal.pgen.1011401 (PMC11729954; doi:10.1371/journal.pgen.1011401)
Supplement: S3 Table — (DOCX) [file pgen.1011401.s007.docx]

**S3 Table.** **Runx3 HCT expressed in clusters of DRG proprioceptive, mechanoceptive or other neuron subtypes in early embryonic development**

| **Runx3 HCT** | | **76 genes expressed in E11.5 proprioceptive clusters 7 and/or 9** |
| --- | --- | --- |
| **Down-regulated**  **(86 genes)** | | *4933431E20Rik, Ablim3, Adcy1, AI593442, Anxa2, Anxa5, Asic1, Cacna2d2, Calca, Camk1g, Cbln2, Ccser2, Cdh12, Cdh18, Cntfr, Cntnap2, Cpeb2, Cyth3, Dock5, Fam189a1, Frmd3, Gabrb3, Gal, Galnt14, Gfra3, Gm20597, Grik1, Grm3, Insrr, Jph4, Kcnb1, Kirrel3, Klhl5, Malat1, March8, Mark1, Mdga1, Mgat4c, Mgst3, Miat, Myo1e, Ncam2, Neurod1, Nfia, Pcdh15, Pde2a, Pfkp, Piezo2, Pirt, Pls1, Ppp1r3b, Prdm12, Prune2, Rab40c, Runx3, Scn7a, Scn10a, Six1, Sema5a, Shisa6, Slc16a2, Slc24a1, Slco4a1, Sorcs1, Spock1, Stra6, Syn2, Syt16, Tbc1d9, Tceanc2, Tenm2, Tnks, Tns1, Usp12, Zdhhc22, Zfp804a* |
|  | |  |
|  | | **24 genes expressed in E11.5 proprioceptive clusters 7 and/or 9** |
| **Up-regulated**  **(158 genes)** | | *Akap8l, Alox5ap, Ap2s1, Arl4c, B4galnt1, Fam129a, Gadd45g, Hist3h2ba, Kit, March1, Mrpl43, Ndufa6, Olfm2, Pctp, Pebp1, Rgs2, Slc1a2, Stk32b, Tox, Tsc22d1, Upf1, Yaf2, Zfhx3, Zfp536* |
|  |  |  |
|  | **8 genes expressed in E11.5 mechanoceptive cluster 11 and/or E12.5 cluster10** |  |
| **Up-regulated**  **(158 genes)** | *Cartpt, Gfra2, Lgmn, Nr5a2, Ntrk2, Pou6f2, Sgk1, Tmem108* |  |
|  |  |  |
|  | **64 genes expressed in E10.5 clusters 5 and/or 6** |  |
| **Up-regulated**  **(158 genes)** | *Aldh16a1, Ap2s1, Arap3, Asf1b, Bop1, Brca1, Cbx1, Ccdc86, Cdc6, Cdca7,* *Cdca8,* *Clns1a, Dusp6, Ect2, Fam198b, Fgfr3, Fip1l1, Fuca1, Gfra2, Gusb, Hapln1, Heatr5a, Hmga2, Hmmr, Hnrnpm, Ifngr1, Iqgap2, Itgb5, Kit, Klk8, Laptm5, Lgmn, Limch1, Mcm3, Mcm5, Meis1, Mn1, Mrpl43, Mtr, Ndufa6, Nxt1, Pctp, Pdzrn3, Pebp1, Peg10, Phb, Plcg2, Prkcq, Pros1, Prtg, Psmd8, Rrm2, Sgk3, Sh3bp1, Snrpa, Sox6, Spcs2, Specc1, Stk17b, Tnfaip8, Upf1, Vps72, Yaf2, Zfp36* |  |

Runx3 HCT that are expressed in proprioceptive clusters 7 and/or 9 at E11.5 or in mechanoceptive cluster 11 at E11.5 and/or cluster 10 at E12.5 or in E10.5 clusters 5 and/or 6, from Faure et al 2020 [1].

1. Faure L, Wang Y, Kastriti ME, Fontanet P, Cheung KKY, Petitpre C, et al. Single cell RNA sequencing identifies early diversity of sensory neurons forming via bi-potential intermediates. Nat Commun. 2020;11(1):4175-89. Epub 2020/08/23. doi: 10.1038/s41467-020-17929-4. PubMed PMID: 32826903; PubMed Central PMCID: PMCPMC7442800.
